# The effectiveness of treatment for depression/depressive symptoms in adults with cancer: a systematic review

**DOI:** 10.1038/sj.bjc.6602949

**Published:** 2006-02-07

**Authors:** S Williams, J Dale

**Affiliations:** 1 Division of Health in the Community, Centre for Primary Health Care Studies, Warwick Medical School, University of Warwick, Coventry CV4 7AL, UK

**Keywords:** depression, systematic review, psychotherapeutic interventions, pharmacological interventions

## Abstract

Depression is common in cancer patients, and this often remains undetected and untreated. Depression has been associated with poorer quality of life, in addition to increased impairment of immune response and poorer survival in cancer patients. Previous systematic reviews and meta-analyses of the efficacy of interventions for cancer patients with depression have failed to distinguish between caseness for depression and depressive symptoms. The findings from this systematic review show that there is limited trial data on the efficacy of prescribed antidepressants in reducing the incidence of major depression and depressive symptoms in cancer patients. Contrary to previous reviews that failed to distinguish between depressive symptoms and depression, this review found very little data from clinical trials (without the possibility of confounding factors) to demonstrate that psychotherapeutic interventions are effective in reducing depression in cancer patients. A number of small-scale, single-centre trials indicated that psychotherapeutic interventions (especially cognitive behavioural therapy) can have effects on depressive symptoms in cancer patients. However, given the methodological limitations of studies to date, lack of evidence should not be interpreted as implying lack of efficacy. In conclusion, there is a need for adequately powered studies of pharmacological and psychotherapeutic studies, which are targeted at cancer patients with a diagnosis of depression and include monitoring of the use of other pharmacological/psychotherapeutic and complementary and alternative medicine interventions.

Studies have reported up to 58% of cancer patients as having depressive symptoms and up to 38% as having major depression (Massie, 2004). Depression may be particularly difficult to detect in patients suffering from cancer, especially those with terminal illness, and is difficult to distinguish from ‘appropriate sadness’ related to cancer diagnosis, treatment and the approach of end of life (Lloyd-Williams, 2000; Bailey *et al*, 2005). There are also difficulties in deciding which somatic symptoms may be attributable to cancer and its consequences, and which may be due to depression (Lloyd-Williams, 2001; Bailey *et al*, 2005). Psychological distress, including adjustment problems, anxiety and depression, typically occurs at many points along the cancer trajectory, and may be exacerbated by physical pain, the effects of treatment, family difficulties, financial worries, etc. The importance of detecting and treating depressive illness in cancer patients lies not only in the relief of psychological distress and its impact on quality of life but also on consequent health service and societal costs. In addition, depression has been associated with increased impairment of immune response (Andersen *et al*, 1998; Newport and Nemeroff, 1998; Reiche *et al*, 2004) and poorer survival (Buccheri, 1998; Faller *et al*, 1999; Watson *et al*, 1999; Faller and Bulzebruck 2002; Herjl *et al*, 2003; Goodwin *et al*, 2004).

Psychosocial needs are often inadequately addressed by cancer services, and depression is frequently unrecognised (Newport and Nemeroff, 1998; Passik *et al*, 1998; Petito and Evans, 1998; Lloyd-Williams, 2000; Sharpe *et al*, 2004; Somerset *et al*, 2004). Clinical practice guidelines for the psychosocial care of cancer patients are available in some countries, such as in the USA and Australia (Turner *et al*, 2005). The National Institute for Clinical Evidence guidelines for the management of depression in primary and secondary care in the UK propose that screening for depression should be undertaken in primary-care and general hospital settings for high-risk groups, which include those with significant physical illnesses (NICE, 2004).

There have been three recent systematic reviews (Barsevick *et al*, 2002; Newell *et al*, 2002; Uitterhoeve *et al*, 2004) and two meta-analyses (Devine and Westlake, 1995; Sheard and Maguire, 1999) of psychotherapeutic interventions for patients with cancer and depression/depressive symptoms, the results of which provide broad support for such interventions. In their meta-analysis of 98 studies, Devine and Westlake (1995) concluded that psychoeducational care is of benefit to adults with cancer and depression. Likewise, Barsevick *et al*'s (2002) systematic review of 36 studies concluded that psychoeducational interventions reduce depressive symptoms in patients with cancer, and that behaviour therapy or counselling alone or in combination with cancer education is beneficial. However, Sheard and Maguire (1999) in their meta-analysis of 20 trials concluded that preventative psychological interventions in cancer patients do not have a clinical effect upon depression. Based on a systematic review of 15 randomised controlled trials, Newell *et al* (2002) made tentative recommendations about the medium-term benefit of group therapy and the long-term benefits of education and structured counselling. Uitterhoeve *et al’*s (2004) systematic review of 13 trials concluded that psychosocial interventions had positive effects on patients with advanced cancer and depression.

In addition, a meta-analysis by Meyer and Mark (1995) reported on the effects of psychosocial interventions with adult cancer patients in terms of emotional adjustment, which involved measures of such constructs as mood state, fear and anxiety, depression, denial or repression, self-esteem and distress. Although the study did not present findings on efficacy exclusively in terms of depression/depressive symptoms, it found that psychosocial interventions have positive effects on emotional adjustment. There were no significant differences found between types of interventions (behavioural interventions, nonbehavioural counselling and therapy, informational and educational methods, organised social support provided by other patients and other nonhospice interventions). Even so, the authors stated that it would be premature to conclude that there were no differences between treatment categories given the possible confounds.

However, none of these systematic reviews and meta-analyses distinguished between the presence of depressive symptoms and caseness for depression in cancer patients, so limiting their applicability to everyday clinical practice.

There have been no systematic reviews or meta-analyses to date published on the efficacy of antidepressant treatments for cancer patients with depression.

The aim of the following study, therefore, was to systematically review the efficacy of psychotherapeutic and antidepressant interventions for cancer patients with depression/depressive symptoms in terms of (i) reduction in depressive symptoms, (ii) reduction in caseness of clinical depression and (iii) adverse effects.

## MATERIALS AND METHODS

We undertook a systematic review of randomised controlled trials of pharmacological and psychotherapeutic interventions for cancer patients with depression/depressive symptoms. The method was based on established guidelines for conducting systematic reviews (Chalmers and Haynes, 1994; Mulrow, 1994; Oxman, 1994).

### Search strategy

References were retrieved by manual searches and through searching electronic databases. The following electronic databases were searched for years 1995–2005 for psychotherapeutic interventions and 1960–2005 for pharmacological interventions: PubMed, CINAHL, Cochrane Library databases DARE, CDSR, CCTR and PsycARTICLES. Manual searches were conducted and relevant references retrieved from those listed in key papers, reports, theses and dissertations. Box 1 provides the search strategy terms.

### Inclusion criteria

The criteria for selecting studies were randomised controlled trials of pharmacological and psychotherapeutic interventions for depression in cancer patients, published in English. Participants were either adult cancer patients with depression or depressive symptoms receiving a pharmacological or psychotherapeutic intervention for depression/depressive symptoms. Studies investigating the efficacy of psychotherapeutic interventions in the presence of pharmacological therapy (or *vice versa*) were excluded, as were those evaluating the efficacy of complementary and alternative (CAM) medicine (including meditation) or information/education strategies.

### Data extraction and synthesis

Data extraction was completed independently by the reviewers (SW) and (JD) and checked through for accuracy. Data were gathered using a data extraction form (Appendix A). The main outcomes were either depressive symptoms or diagnosed clinical depression measured by a separate scale or as part of a composite outcome measure. Study quality was assessed with the Methodological Quality Instrument (Appendix A) developed by Cho and Bero (1994). Studies were assessed as being of low methodological quality if they failed to meet the minimum requirements on each of the following aspects of study design: adequacy of sample size, randomisation, blinding, method of allocation concealment, clear description of treatment, representative source of subjects, use of diagnostic criteria, number of and reasons for withdrawal from intervention and how dealt with in the analysis, outcome measures described clearly, use of validated instruments, and steps taken to control for possible confounding factors.

## RESULTS

### Study inclusion and characteristics

**Figure 1:**
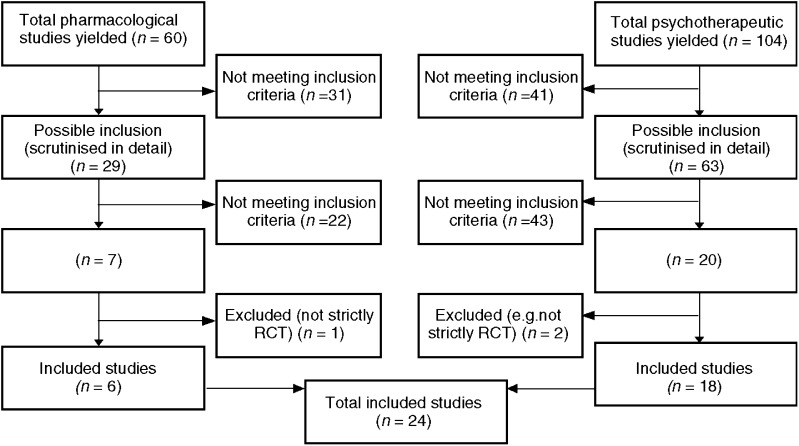
Number of studies yielded by search strategy.

Figure 1 shows the numbers of studies yielded by the search strategy. In all, 29 papers reporting pharmacological studies and 63 reporting psychotherapeutic interventions were identified as potentially relevant and were carefully read. Of these, we excluded 65 because they did not meet the inclusion criteria, and a further two were excluded because they were not strictly randomised controlled trials (Holland *et al*, 1998; Kissane *et al*, 2003) and one because it failed to measure baseline levels of depressive symptoms (Stiegelis *et al*, 2004). This left a total of 24 studies (Table 1), of which six were trials of pharmacological treatments and 18 were of psychotherapeutic interventions.

### Pharmacological studies

The six pharmacological studies were randomised placebo-controlled trials conducted in the United States of America (*n*=4) (Musselman *et al*, 2001; Fisch *et al*, 2003; Morrow *et al*, 2003; Roscoe *et al*, 2005) and Belgium (*n*=2) (Razavi *et al*, 1996; Van Heeringen and Zivkov, 1996). The mean sample size of intervention and control groups was 83 (range 20–277 patients) and 83 (range 20–272 patients), respectively. The average age of patients ranged from 50 to 61 years. Between half to 100% of participants were female. Three studies were multicentre trials (Razavi *et al*, 1996; Fisch *et al*, 2003; Morrow *et al*, 2003) and three were single-centre trials (Van Heeringen and Zivkov, 1996; Musselman *et al*, 2001; Roscoe *et al*, 2005).

#### Methodological quality

The methodological quality of these trials is summarised in Table 2. The sample size exceeded 100 patients in two of the six (33%) trials (Fisch *et al*, 2003; Morrow *et al*, 2003). The method of randomisation to groups was sufficiently well described in all studies, but the method of allocation concealment was adequately described in only four trials (Razavi *et al*, 1996; Musselman *et al*, 2001; Fisch *et al*, 2003; Roscoe *et al*, 2005). Blinding of investigators was reported in five trials (Razavi *et al*, 1996; Van Heeringen and Zivkov, 1996; Musselman *et al*, 2001; Fisch *et al*, 2003; Roscoe *et al*, 2005) and blinding of subjects in all trials. All studies provided a clear description of the intervention, had a representative source of subjects and provided the number and reasons for withdrawals. Dropout rates for intervention and controls ranged from 0 to 56%. Three trials stated that intention-to-treat analyses were used to deal with patients who were lost to follow-up (Razavi *et al*, 1996; Van Heeringen and Zivkov, 1996; Musselman *et al*, 2001).

Two trials selected subjects on the basis of a depressive disorder using Diagnostic and Statistical Manual of Mental Disorders, third edition (DSM-III) criteria (Razavi *et al*, 1996; Van Heeringen and Zivkov, 1996); one selected patients who were to receive high-dose alpha therapy (which has been associated with symptoms of major depression) (Musselman *et al*, 2001). Three trials reported on the efficacy of the pharmacological treatment in terms of caseness for depression (Razavi *et al*, 1996; Musselman *et al*, 2001; Roscoe *et al*, 2005) with the other three trials reporting on change in the level of depressive symptoms as indicated by scores on questionnaires (Van Heeringen and Zivkov, 1996; Fisch *et al*, 2003; Morrow *et al*, 2003). All outcome measures were clearly described and were valid and reliable (Table 1).

Five trials investigated the efficacy of selective serotonin reuptake inhibitors with three investigating paroxetine (Musselman *et al*, 2001; Morrow *et al*, 2003; Roscoe *et al*, 2005) and two fluoxetine (Razavi *et al*, 1996; Fisch *et al*, 2003), while the remaining study investigated the tricyclic antidepressant mianserin (Van Heeringen and Zivkov, 1996). All six trials avoided/monitored pharmacological cointerventions by trial design. None of the six studies reported avoiding or monitoring the use of potentially confounding psychotherapeutic or CAM cointerventions by subjects during the study periods.

#### Effectiveness and tolerability

Depression: One trial found paroxetine effective in reducing major depression in cancer patients with malignant melanoma who were to receive high-dose interferon alpha therapy (Musselman *et al*, 2001). Major depression developed in 11% (two of 18) of the paroxetine and 45% (nine of 20) of the placebo group, and 5% of paroxetine compared to 35% of the placebo group had to discontinue interferon alpha because of severe depressive distress. In terms of tolerability, retinal haemorrhages developed in three patients (severely in one patient) who were taking paroxetine. The sample size was only 40 patients, but no subjects withdrew from the study.

Another trial found paroxetine to be effective in reducing caseness for depression in breast cancer patients receiving chemotherapy (Roscoe *et al*, 2005). At final follow-up (cycle 4 of chemotherapy), only four of the original 13 patients (31%) in the paroxetine group who had baseline depression (scoring greater than 19 on the Center for Epidemiological Studies Depression scale) had scores above the cutoff, while all 13 (100%) of the initially depressed patients in the placebo group remained above the threshold. Dropout rates were comparable for intervention and controls (25 and 21%, respectively).

Fluoxetine was not effective in reducing caseness for depression in a trial that included patients with breast, gynaecological or haematological cancer who presented with major depressive disorder (Razavi *et al*, 1996). The successful response rate (defined by Hospital Anxiety and Depression Scale (HADS) score lower than 8 after 5 weeks of treatment) was not significantly higher (11%) in the intervention group than the placebo group (7%). Side effects between groups were not significantly different, although there was a trend towards digestive and neuropsychiatric adverse events in the intervention group. There were, however, significantly more dropouts from the intervention group (33%) compared with controls (15%) (*P*=0.04).

Depressive symptoms: Paroxetine was found to be effective in reducing depressive symptoms in breast, lung, haematological, gynaecological and gastrointestinal cancer patients who reported fatigue at their second chemotherapy cycle (Morrow *et al*, 2003). Dropout rates were comparable for intervention and controls (12 and 14%, respectively). Paroxetine was also found to be effective in reducing depressive symptoms in breast cancer patients undergoing chemotherapy (Roscoe *et al*, 2005).

Fluoxetine was found to be effective in reducing depressive symptoms in patients with advanced solid tumours (Fisch *et al*, 2003). Frequency of vomiting was significantly higher in patients receiving fluoxetine (nine of 27 (33%)) compared with patients receiving placebo (two of 43 (4.6%)), but there were significantly more fluoxetine patients receiving radiation therapy. Dropout rates were high with 54% for the intervention group and 44% for the controls.

There was a reduction in depressive symptoms in breast cancer patients who received mianserin compared with those receiving placebo (Van Heeringen and Zivkov, 1996). Tolerability appeared to be good with no significant differences between groups for adverse events and changes in vital signs. There were also significantly fewer dropouts from the intervention group (21%) when compared to controls (56%) (*P*=0.014).

### Psychotherapeutic studies

Characteristics of the 18 included trials of psychotherapeutic interventions are shown in Table 1. Studies were conducted in Europe (*n*=5) (Burton *et al*, 1995; Marchioro *et al*, 1996; McArdle *et al*, 1996; Moynihan *et al*, 1998; Kuijer *et al*, 2004), the United States of America (*n*=8) (Evans and Connis, 1995; Sandgren *et al*, 2000; Antoni *et al*, 2001; Classen *et al*, 2001; Rawl *et al*, 2002; Winzelberg *et al*, 2003; Given *et al*, 2004; Weber *et al*, 2004) Canada (*n*=1) (Goodwin *et al*, 2001), Australia (*n*=3) (Edelman *et al*, 1999; McLachlan *et al*, 2001; Petersen and Quinlivan, 2002) and Japan (*n*=1) (Fukui *et al*, 2000). The mean sample size in the intervention and control group was 61 patients (range 15–296) and 52 patients (range 15–154), respectively. The average age of patients ranged from 49 to 64 years. The proportion of female participants ranged from between 41 to 100%. Eight studies were multicentre trials (McArdle *et al*, 1996; Classen *et al*, 2001; Goodwin *et al*, 2001; McLachlan *et al*, 2001; Petersen and Quinlivan, 2002; Rawl *et al*, 2002; Given *et al*, 2004; Weber *et al*, 2004) and 10 were single-centre trials (Burton *et al*, 1995; Evans and Connis, 1995; Marchioro *et al*, 1996; Moynihan *et al*, 1998; Edelman *et al*, 1999; Fukui *et al*, 2000; Sandgren *et al*, 2000; Antoni *et al*, 2001; Winzelberg *et al*, 2003; Kuijer *et al*, 2004).

#### Methodological quality

The methodological quality of these trials is summarised in Table 2. The sample size exceeded 100 in eight of 18 (44%) trials (Burton *et al*, 1995; McArdle *et al*, 1996; Edelman *et al*, 1999; Classen *et al*, 2001; Goodwin *et al*, 2001; McLachlan *et al*, 2001; Rawl *et al*, 2002; Given *et al*, 2004). All studies randomly allocated subjects to treatment groups. Blinding of investigators was reported in two (11%) trials (Moynihan *et al*, 1998; Goodwin *et al*, 2001). All 18 (100%) studies provided a clear description of the intervention and had a representative source of subjects. Reporting of attrition of subjects was provided in 16 (89%) studies and reasons for withdrawal in 13 (72%) (see Table 1). Dropout rates for intervention and controls ranged from 0 to 41%.

In all, 17 (94%) studies were preventative, with subjects selected on the basis of a diagnosis of cancer and only one study selected patients with a diagnosis of depression according to DSM-III criteria (Evans and Connis, 1995). All outcome measures were clearly described and reliable. Only two (11%) studies avoided some psychotherapeutic cointerventions by trial design (Goodwin *et al*, 2001; Weber *et al*, 2004). None of the trials reported avoiding or monitoring the use of pharmacological or CAM cointerventions.

#### Depression

Only four of 18 (22%) trials reported the efficacy of psychotherapeutic interventions in terms of caseness for depression (Burton *et al*, 1995; Moynihan *et al*, 1998; McLachlan *et al*, 2001; Petersen and Quinlivan, 2002). Of these, one found counselling/psychotherapy to be effective in reducing caseness for depression in breast cancer patients at 1 year follow-up (*P*=0.037; Burton *et al*, 1995), and another found counselling/relaxation to be effective in reducing caseness for depression in gynaecological cancer at 6 weeks follow-up (*P*=0.0001; Petersen and Quinlivan, 2002). Computer-based assessments and individually tailored care plans were also associated with a reduction in the proportion of moderately or severely depressed cancer patients at 2 and 6 months follow-up (McLachlan *et al*, 2001); the statistical significance of this finding was not reported. However, in men newly diagnosed with testicular cancer, cognitive behaviour therapy/problem-solving therapy was not found to significantly reduce the proportion scoring above threshold for depression (Moynihan *et al*, 1998).

#### Depressive symptoms

All 18 trials (100%) reported the impact of psychotherapeutic interventions on the level of depressive symptoms as indicated by scores on questionnaires.

#### Cognitive behavioural therapy

Several trials reported cognitive behavioural therapy (CBT) to be effective. CBT was found to reduce depressive symptoms in patients with breast, gastrointestinal, lymphoma, brain and lung cancers at 1 week postintervention (ES=0.55) (Kuijer *et al*, 2004), and also in depressed patients with lung, bladder, prostate and head–neck cancers receiving radiation treatment at 8 weeks follow-up (*P*<0.01) (Evans and Connis, 1995). Efficacy was also reported for women with metastatic breast cancer immediately after completion of the intervention (*P*=0.008) (Edelman *et al*, 1999); women newly treated for stage 0–II breast cancer at 3 months postintervention, 6 and 12 months (*P*<0.04) (Antoni *et al*, 2001); women with metastatic breast cancer assessed for depressive symptoms at 4, 8 and 12 months (ES=0.27) (Classen *et al*, 2001); and in metastatic breast cancer at 1 year (*P*=0.002) (Goodwin *et al*, 2001). In addition, one study found CBT to have a short-term (10 weeks), but no long-term (20 weeks), effect on depressive symptoms in patients diagnosed with a solid tumour and receiving a first cycle of chemotherapy (ES=0.25 and 0.33 at 10 and 20 weeks, respectively) (Given *et al*, 2004). However, patients in the experimental group who entered with higher depressive symptoms had higher levels of depressive symptoms at 10 weeks than patients in the control group. Two other trials found CBT to have no significant effects on depressive symptoms in two studies of women with breast cancer (Fukui *et al*, 2000; Sandgren *et al*, 2000), but these both had sample sizes below 100 as compared to only two of the six studies that found CBT to be effective.

#### Counselling/psychotherapy

Counselling/psychotherapy was found to be effective in reducing depressive symptoms in newly diagnosed women with nonmetastatic breast cancer assigned to adjuvant chemotherapy after surgery, with persistence over 9 months follow-up (ES=0.27, Marchioro *et al*, 1996). Counselling/relaxation was also found to be effective in reducing mild/moderate depressive symptoms 6 weeks after therapy in patients with gynaecological cancer (HADS mild/moderate depression subscale, *P*=0.02) but not severe levels of depressive symptoms (GHQ-28 Major depressive symptoms subscale, NS; Petersen and Quinlivan, 2002).

#### Supportive

The provision of group social support was found to be as effective as CBT in reducing depressive symptoms in depressed patients with lung, bladder, prostate and head–neck cancer who were receiving radiation treatment, when assessed at 8 weeks (*P*<0.01) and also effective at 6 months follow-up compared with controls (*P*<0.01) (Evans and Connis, 1995). Peer support from long-term survivors of prostate cancer was found to be effective in reducing depressive symptoms in men who had recently undergone a radical prostatectomy when assessed at 4 weeks (*P*=0.02), but not at 8 weeks, follow-up (Weber *et al*, 2004).

A web-based social support group was also found to be effective in reducing depressive symptoms in women with primary breast cancer when assessed at the end of the 12 weeks intervention (ES=0.54) (Winzelberg *et al*, 2003).

In a complex trial that compared different levels of support, routine care plus education/information/counselling/support from breast care nurse was found to be more effective than (1) routine care plus education/information/counselling/support from a voluntary organisation, (2) routine care plus education/information/counselling/support from breast care nurse and voluntary organisation or (3) routine care from ward staff (*P*=0.015, 0.003, 0.072; McArdle *et al*, 1996). The failure to reduce morbidity in the combined group is difficult to explain.

Computer-based assessments and individually tailored care plans together with emotional support and counselling by nurses was found to reduce depressive symptoms midway through the intervention in newly diagnosed breast, colon or lung cancer patients who were receiving chemotherapy (*P*=0.05) (Rawl *et al*, 2002). Computer-based assessments and individually tailored care plans were also found to reduce depressive symptoms (*P*=0.001) at 6 months follow-up for patients with lung, head and neck, gynaecologic, haematology/lymphoma, melanoma and other types of cancers unspecified in the paper (McLachlan *et al*, 2001).

## DISCUSSION

This systematic review indicates that there is limited trial data on the efficacy/tolerability of antidepressants and psychotherapeutic interventions for patients with cancer and depression. The reviewed studies varied in the type of pharmacological or psychotherapeutic interventions employed, the characteristics of the studied populations, the type, grade and stage of subjects’ cancer, the treatments being received, and the trial design, including outcome measures used. Most were of small size and lacked control for possible confounding factors. Such limitations indicate the need for cautious interpretation of the review's findings beyond the contexts within which these studies were conducted.

### Pharmacological studies

Only three of the six trials that involved pharmacological interventions reported the efficacy of antidepressants in terms of change in caseness for clinical depression as opposed to change scores indicating levels of depressive symptoms. Paroxetine was found to be effective in reducing major depression in patients with malignant melanoma who were to receive high-dose interferon alpha therapy (Musselman *et al*, 2001), and in reducing caseness for depression in breast cancer patients receiving chemotherapy (Roscoe *et al*, 2005). Fluoxetine was not effective in reducing caseness for depression in a trial that included patients with breast, gynaecological or haematological cancer (Razavi *et al*, 1996). However, the study by Razavi *et al* (1996) was only of brief duration (5 weeks) and higher doses of fluoxetine were not used.

Paroxetine and fluoxetine were both effective in reducing depressive symptoms in three trials that included patients with a range of cancers (breast, lung, haematological, gynaecological and gastrointestinal) (Fisch *et al*, 2003; Morrow *et al*, 2003; Roscoe *et al*, 2005), and the tetracyclic antidepressant mianserin was also shown to be effective in reducing depressive symptoms in breast cancer (Van Heeringen and Zivkov, 1996).

Some nonpsychological benefits of antidepressant therapy also emerged, such as improved adherence to cancer treatment. For example, paroxetine significantly decreased the likelihood that interferon alpha therapy in malignant melanoma would be discontinued because of severe depression or related neurotoxic effects (Musselman *et al*, 2001).

Although tolerability was only reported in four of the six pharmacological studies, overall, the tolerability of antidepressants in patients with cancer appears to be good. Although there was some evidence of adverse effects, these may well have been caused by other aspects of the treatment (e.g. radiotherapy, interferon alpha) that these patients were receiving.

### Psychotherapeutic interventions

Only four of the reviewed studies reported the efficacy of psychotherapeutic interventions in treating depression. Of these, two trials reported significant benefits of counselling/psychotherapy (Burton *et al*, 1995) and counselling/relaxation (Petersen and Quinlivan, 2002) in reducing caseness for depression for patients with breast cancer and gynaecological cancer, respectively, and a third found computer-based assessment (including completion of the Beck Depression Inventory) and individually tailored care plans by a nurse to be associated with a reduction in the proportion of patients with moderate or severe depression (McLachlan *et al*, 2001).

There is more extensive evidence that psychotherapeutic interventions are effective in reducing depressive symptoms in cancer patients, at least in the short-term. Seven trials found cognitive behavioural therapy to be effective in reducing depressive symptoms, with persistence of improvement demonstrated for up to a year (Evans and Connis, 1995; Edelman *et al*, 1999; Antoni *et al*, 2001; Classen *et al*, 2001; Goodwin *et al*, 2001; Given *et al*, 2004; Kuijer *et al*, 2004). However, two trials found CBT to have no significant effects on depressive symptoms (Fukui *et al*, 2000; Sandgren *et al*, 2000), and one study found CBT to have a short-term (10 weeks), but no long-term (20 weeks), effect (Given *et al*, 2004).

Other psychotherapeutic approaches that may be effective in lowering depressive symptoms include supportive interventions in the form of social support groups, supportive dyads or web-based support groups (Evans and Connis, 1995; Winzelberg *et al*, 2003; Weber *et al*, 2004), computer-based assessments and individually tailored care plans (McLachlan *et al*, 2001; Rawl *et al*, 2002), and counselling/psychotherapy and counselling/relaxation (Burton *et al*, 1995; Marchioro *et al*, 1996).

### Methodological limitations

A major limitation of all 24 trials reviewed was the lack of consistent avoidance/monitoring of the use of psychotherapeutic, pharmacological or CAM cointerventions by subjects. In general the reviewed studies had small sample sizes, and had not attempted to control for confounding, so limiting the validity of findings. Many patients being treated for cancer will be receiving care from a variety of primary-, secondary- and tertiary-care clinicians. Many patients also make use of CAM to manage their condition (Tatsumura *et al*, 2003; McClain-Jacobson *et al*, 2004; Montazeri *et al*, 2005), and there are CAM approaches associated with decreasing depression/depressive symptoms in cancer patients (Targ and Levine, 2002; Fellowes *et al*, 2004; Cohen *et al*, 2005; Rajasekaran *et al*, 2005). Such confounding factors can be controlled for at the stage of data analysis, but this requires adequate numbers of subjects to have been recruited into trials and data on cointerventions to have been collected. While this review was limited to non-CAM interventions, the same need for rigour and control of confounding factors would apply to this body of research.

A large proportion of the psychotherapeutic studies were single-centre trials, so limiting the generalisability of findings beyond the context of the trial setting. Psychotherapeutic interventions are likely to be highly dependent on the practitioners’ training, skills and other attributes. Hence, multicentre trials are needed to confirm the applicability and effectiveness of interventions. Furthermore, many of the studies report findings that only just achieve statistical significance, suggesting the likelihood of publication bias that needs to be considered in the interpretation of their findings.

There was only one trial that selected patients on the basis of a diagnosis of depression (Evans and Connis, 1995), but the change in caseness for depression was not reported in this study. The negative findings of some studies may reflect that recruited subjects did not have significant psychological morbidity; psychotherapeutic and pharmacological interventions should be offered to patients with clinically meaningful levels of depression/depressive symptoms.

Given the small number of studies on the efficacy on antidepressants with cancer patients in general, there is a need for further work with specific groups of cancer patients. Tolerability data were not always recorded and reported. It is important that future studies include recording and reporting of adverse effects, especially as such effects are likely to have an impact on compliance (Stokes, 1993). Concerns that antidepressants may lead to or accelerate the development of cancer also need further investigation (Brandes *et al*, 1992; Wallace *et al*, 2001).

Finally, while the randomised controlled studies in this review may be of relevance to palliative care, no studies were conducted with palliative care patients as subjects. Controlled studies including such subjects are even more difficult to conduct than those on nonpalliative cancer patients. Depression is difficult to diagnose in such patients, and depressive symptoms are very similar to the general symptoms of end-stage cancer. While high attrition rates, together with high heterogeneity, would lead to the need for very large sample sizes, recruitment is difficult for practical and ethical reasons (Grande and Todd, 2000; Addington-Hall, 2002). There is also a lack of reporting on syndromal depression and this too requires further work.

## CONCLUSION

The sparse number of studies of pharmacological interventions for cancer patients with depression provides some evidence that antidepressants are effective in reducing depression/depressive symptoms in cancer patients. Although more data are needed regarding the safety and efficacy of antidepressants, there is some evidence that cancer patients with depression are responsive to treatment. Overall, the small number of trials of pharmacological interventions for cancer patients with depression/depressive symptoms, high dropout rates in some trials and lack of reporting of adverse events/tolerability should caution against drawing definitive conclusions about which antidepressants are most effective or well tolerated by cancer patients in general or by patients with specific types of cancer.

There is limited trial data on the efficacy of psychotherapeutic interventions in treating depression/depressive symptoms in cancer patients. Cognitive behavioural therapy appears to be effective in reducing depressive symptoms in cancer patients. Social support for cancer patients may also be effective in reducing depressive symptoms.

However, there is a need for more rigorous investigation of the efficacy of both pharmacological and psychotherapeutic interventions, including avoidance/monitoring of the confounding factors of the use of other pharmacological/psychotherapeutic and CAM cointerventions by subjects. In addition, there is also a need for studies comparing the efficacy of psychotherapeutic *vs* pharmacological interventions. With the known side effects and the controversies about the possible stimulation of malignant growth by antidepressant drugs, there is a need for careful and rigorous investigation of the efficacy of pharmacological treatment for depression in cancer patients and more importantly of possible psychotherapeutic alternatives (Wallace *et al*, 2001).

Finally, the current lack of clinical trial data to robustly demonstrate efficacy is not synonymous with evidence of ineffectiveness. It would be entirely inappropriate to use the findings of this review to deny patients with cancer access to treatment for depression and depressive symptoms because of a lack of trial data on effectiveness. The review indicates that there remains a pressing need for more thorough and extensive investigation of the effectiveness and consequences of different approaches to managing depression in cancer patients to inform the design and delivery of effective healthcare services.

## Figures and Tables

**Table 1:** Characteristics of included studies (*n*=24)

	**Subjects**	**Intervention (*n*)**			
**Author (date), country of origin and study quality**	**I=Intervention C=Control**	**I=Intervention C=Control**	**Outcome measures for depression or depressive symptoms**	**Results**	**Caseness for depression**
*Pharmacological studies (cancer various sites)*					
Morrow *et al* (2003), USA Design: Randomised placebo-controlled trial Random allocation to treatment groups: YES Concealed treatment allocation: UNCLEAR Blinding of investigators: NO Blinding of subjects: YES Known confounders accounted for by study design: PARITIAL Attrition of subjects and reasons: YES Dropout rate: I=12%, C=14%	549 cancer patients with a diagnosis of any type of cancer reporting fatigue at their second chemotherapy cycle Mean age (years) (s.d.): I=56.5 (12.6) C=56.3 (12.3) Gender: I=80% female C=71% female	I=**SSRI paroxetine** (20 mg day^−1^) for 8 weeks (*n*=277) C=placebo for 8 weeks (*n*=272)	Depressive symptoms CES-D and DD Assessments performed at baseline (cycle 2 of chemotherapy), cycle 3 and cycle 4	At conclusion (cycle 4) the paroxetine group had a significantly lower mean level of depression than placebo group as measure on the CES-D (*P*=0.003) and DD (*P*<0.001) Mean decrease in CES-D score from baseline to end point was 18.9% in the paroxetine group and 6.3% in the control group Tolerability: not reported	Not reported

Fisch *et al* (2003), USA Design: Randomised placebo-controlld trial Random allocation to treatment groups: YES Concealed treatment allocation: YES Blinding of investigators: YES Blinding of subjects: YES Known confounders accounted for by study design: PARTIAL Attrition of subjects and reasons: YES Dropout rate: I=54%, C=44%, NS	163 patients with an advanced solid tumour and expected survival between 3 and 24 months Mean age (years): I=61 C=59 Gender: I=45% female C=55% female	I=**SSRI fluoxetine** (20 mg day^−1^) for 12 weeks (*n*=83) C=placebo for 12 weeks (*n*=80)	Depressive symptoms: 11-item BZSDS Assessments performed at baseline and every 3 to 6 weeks thereafter. Patients were assessed for 12 weeks and complete assessment involved three to five sessions of data collection. After 12 weeks, patients were given option to continue the study drug blinded for up to 9 months	The fluoxetine group improved significantly compared with placebo on depression scale (*P*=0.0005) Mean decrease in BZSDS score from baseline to end point was 13.5% in the fluoxetine group and 2.4% in the control group Tolerability: 9 (33%) of 27 patients in fluoxetine arm reported one or more episodes of emesis at study completion visit compared with two (4.6%) of 43 patients receiving placebo (*P*=0.01) although there were significantly more fluoxetine patients receiving radiation therapy	Not reported

*Pharmacological studies (cancer various sites)*					
Razavi *et al* (1996), Belgium Design: Experimental randomised placebo-controlled trial Random allocation to treatment groups: YES Concealed treatment allocation: YES Blinding of investigators: YES Blinding of subjects: YES Known confounders accounted for by study design: PARTIAL Attrition of subjects and reasons: YES Dropout rate: I=33%, C=15% (*P*=0.04)	91 cancer patients with a major depressive disorder or an adjustment disorder as defined by DSM-III Patients with score of 13 or higher on the HADS at start Mean age (years) (s.d.): I=53.2 (11.4) C=52.6 (11.3) Gender: I=77% female C=82% female	After a single-blind placebo period of 1 week patients (to exclude early placebo responders and false-positive cases for depression) were randomised to: I=**SSRI** **fluoxetine** treatment (20 mg day^−1^) (*n*=45) C=Placebo (*n*=46)	Depression/depressive symptoms MADRS, HADS Measured at baseline, 1, 3 and 5 weeks after the intervention	HADS, NS Tolerability: Frequencies of side effects between groups was not significantly different Digestive and neuropsychiatric types of adverse events were more frequent in the intervention group although this was not significant	Caseness, NS: The successful response rate defined by HADS score lower than 8 after 5 weeks of treatment was not significantly higher (11%) in the intervention group than the placebo group (7%)

*Pharmacological studies (skin cancer)*					
Musselman *et al* (2001), USA Design: Randomised placebo-controlled trial Random allocation to treatment groups: YES Concealed treatment allocation: YES Blinding of investigators: YES Blinding of subjects: YES Known confounders accounted for by study design: PARTIAL Attrition of subjects and reasons: YES Dropout rate: I=0%, C=0%	40 patients with malignant melanoma who were to receive high-dose interferon alpha therapy Mean age (years) (s.d.): I=52.8 (7.6) C=50.1 (13.4) Gender: I=50% female C=50% female	Interferon alpha has been associated with symptoms that overlap with those found in major depression I=**SSRI paroxetine**: Two weeks before the initiation of interferon alpha therapy SSRI Paroxetine (10 mg day^−1^ 1 tablet) for one week (20 mg day^−1^ 2 tablets) for one week. Four weeks after the initiation of paroxetine therapy the dosage could be increased up to 40 mg day^−1^ 4 tablets) (*n*=20) C=placebo therapy (*n*=20)	Depression: HAM-D21, CDS, DSM-IV Assessment performed at baseline and at regularly scheduled intervals after the initiation of interferon alpha therapy for the first 12 weeks of a planned 48-week treatment period.	Tolerability: After 12 weeks, small reversible retinal haemorrhages developed in two patients and one patient had more severe retinal haemorrhages with associated irreversible loss of vision. All three patients were taking paroxetine. Retinal haemorrhage is a rare side effect of paroxetine and high rates of retinal complications occur in patients treated with interferon alpha. Paroxetine significantly decreased the likelihood that interferon alpha therapy would be discontinued because of severe depression or related neurotoxic effects (*P*=0.03)	Paroxetine treatment significantly reduced the incidence of major depression (*P*=0.04) Symptoms consistent with major depression developed in two of the 18 (11%) patients in paroxetine group and nine of the 20 (45%) patients in the placebo group HAM-D21 (*P*=0.01)

*Pharmacological studies (breast cancer)*					
Roscoe *et al* (2005), USA Design: Randomised placebo-controlled trial Random allocation to treatment groups: YES Concealed treatment allocation: YES Blinding of investigators: YES Blinding of subjects: YES Known confounders accounted for by study design: PARTIAL Attrition of subjects and reasons: YES Dropout rate I=25%, C=21%	94 female breast cancer patients receiving at least four cycles of chemotherapy and not undergoing concurrent radiation or interferon treatments Mean age (years) (s.d.): I=52.2 (9.3) C=52.2 (10.2)	I=**SSRI paroxetine** (20 mg day^−1^) for four cycles of chemotherapy (*n*=44) C=placebo for four cycles of chemotherapy (*n*=50)	Depression/depressive symptoms CES-D and DD	Paroxetine was more effective than placebo in reducing depressive symptoms during chemotherapy as measured by the CES-D (*P*=0.006) and the DD (*P*=0.07) Mean decrease in CES-D score from baseline to end point was 40% in the paroxetine group and 14% in the control group Tolerability: Not reported	By cycle 4, only four of the original 13 patients in the paroxetine group who had baseline CES-D scores greater than 19 still had scores above that cutoff point. This compares to all 13 of the initially depressed patients in the placebo group remaining above threshold.

Van Heeringen and Zivkov (1996), Belgium Design: Experimental randomised placebo-controlled trial Random allocation to treatment groups: YES Concealed treatment allocation: UNCLEAR Blinding of investigators: YES Blinding of subjects: YES Known confounders accounted for by study design: PARTIAL Attrition of subjects and reasons: YES Dropout rate: I=21%, C=56% (*P*=0.014)	55 women with breast cancer (stage I or stage II without metastases) with a diagnosis of depression according to the DSM-III criteria Mean age (years) (s.vnd.): I=51 (8) C=53 (8)	I=**TCA** **mianserin** treatment: 1 week 30 mg day^−1^, then 60 mg day^−1^ (*n*=28) C=Placebo (*n*=27)	Depressive symptoms HAM-D21 Measured at 14, 28 and 42 days after treatment	There were significantly lower HAM-D21 scores for the mianserin group at 28 days (*P*=0.004) and 42 days (*P*=0.004) Tolerability: No significant differences were found between numbers of patients complaining of at least one adverse event at any assessment point. No clinically relevant changes were seen in vital signs	Not reported

*Psychotherapeutic studies (cancer various sites)*					
Kuijer *et al* (2004), The Netherlands Design: Experimental randomised controlled trial Random allocation to treatment groups: YES Concealed treatment allocation: UNCLEAR Blinding of investigators: NO Blinding of subjects: NO Known confounders accounted for by study design: NO, cointerventions not reported Attrition of subjects and reasons: YES Dropout rate: I=37.5%, C=40.7%	59 couples with medical diagnosis of cancer in one partner with an estimated life expectancy of at least 6 months for the ill partner Mean age (years) (s.d.): I=Patient 50 (12) C=Patient 49 (10) Gender patient: I=70% female C=68% female	I=**CBT**: brief counselling program directed at couples focused on the exchange of social support and help between both partners. Five 90 min sessions led by a psychologist held biweekly. The approach was cognitive-behaviourally oriented (*n*=20) C=waiting list group (*n*=19)	Depressive symptoms Psychological distress – CES-D I=Once before intervention, 1 week postintervention and 3 months postintervention I2=twice before intervention, 1 week postintervention and 3 months postintervention	Among patients psychological distress decreased significantly 1 week after the intervention (*P*<0.05), ES=0.55 Mean decrease in CES-D score for patients from baseline to end point was 31.7% in the experimental group and −7.6% in the waiting list group	Not reported

Given *et al* (2004), USA Design: Experimental randomised controlled trial Random allocation to treatment groups: YES Concealed treatment allocation: UNCLEAR Blinding of investigators: NO Blinding of subjects: NO Known confounders accounted for by study design: NO, cointerventions not reported Attrition of subjects and reasons: attrition, but no reasons given Dropout rate: I=32%, C=27%	237 patients diagnosed with a solid tumour and within 56 days of undergoing a first cycle of chemotherapy Mean age (years): I=59.3 C=61.2 Gender: Approx 80% female	I=**CBT**: 10 contact (1 h sessions), 20 week experimental group cognitive behavioural approach for symptom management. Each strategy for addressing a symptom problem was evaluated at follow-up with the patient. If the strategy had been tried and was effective it was retained and if not new strategies were introduced (*n*=118) C=conventional care (*n*=119)	Depressive symptoms – CES-D Baseline, 10 and 20 weeks	The intervention had a short-term (10 weeks) but no long-term (20 weeks) effect on patient depressive symptoms (ES=0.25–0.33 at 10 and 20 weeks, respectively) The intervention was more effective in lowering depressive symptoms at 10 weeks among patients with higher levels of baseline symptom severity. Among patients with high levels of baseline depression, the intervention was less successful in lowering depressive symptoms at 10 weeks than the conventional care control group alone	Not reported

*Psychotherapeutic studies (cancer various sites)*					
Rawl *et al* (2002), USA Design: Experimental randomised controlled trial Random allocation to treatment groups: YES Concealed treatment allocation: YES Blinding of investigators: NO Blinding of subjects: NO Known confounders accounted for by study design: NO, cointerventions not reported Attrition of subjects and reasons: YES Dropout rate: I=38%, C=19%	109 patients newly diagnosed with breast, colon or lung cancer who were receiving chemotherapy Mean age (years) (s.d.): 55.7 (11.9) Gender: I=87% female C=73% female	I=**Computer-based assessment and individually tailored care plans**: A menu-driven computer program that guided clinical assessment, problem identification, selection of interventions and measurement of outcome. For each symptom or problem exhaustive problem-specific lists of appropriate interventions were available. Nurse specialists could input assessments of patients’ physical and psychosocial functioning (including anxiety and depression) and symptom experience. It allowed for individual tailoring of care plans. Nurses also provided emotional support and counselling during each session (*n*=55) C=standard care (*n*=54)	Depressive symptoms CESD-20 Measured at baseline, time 2 midway through intervention and time 3 one month postintervention (24 weeks)	Patients who received the intervention had significantly fewer symptoms between baseline and time 2. CESD-20 at time 2 (*P*=0.05)	Not reported

McLachlan *et al* (2001), Australia Design: Experimental randomised controlled trial Random allocation to treatment groups: YES Concealed treatment allocation: YES Blinding of investigators: NO Blinding of subjects: NO Known confounders accounted for by study design: NO, cointerventions not reported Attrition of subjects and reasons: YES Dropout rate: I=28%, C=31%	450 cancer patients The authors stated that patient demographics were well balanced in the two arms. Median age (years) (range):61 (18–92) Gender: 41% female	I=**Computer-based assessment and individually tailored care plans**: A computer-generated one-page summary of the questionnaire results (CNQ, EORTC QLQ-30, BDI) was made available immediately for consideration during the consultation with the doctor. After discussion with the doctor and patient the co-ordination nurse formulated an individualised management plan (*n*=296) C=conventional clinical encounter (*n*=154)	Depression/depressive symptoms BDI –short form Measured at baseline, 2 and 6 months after intervention	For patient subgroup that were moderately or severely depressed at baseline, significant reduction in depression at 6 months (*P*=0.001)	At 2 and 6 months, 73 and 90% of patients who were moderately or severely depressed at baseline were still so, whereas in the intervention arm, there were 58 and 45%, respectively

*Psychotherapeutic studies (breast cancer)*					
Evans and Connis (1995), USA Design: Experimental randomised controlled trial Random allocation to treatment groups: YES Concealed treatment allocation: NO Blinding of investigators: NO Blinding of subjects: NO Known confounders accounted for by study design: NO, cointerventions not reported Attrition of subjects and reasons: YES Dropout rate: 8% overall	78 stage II depressed cancer patients receiving radiation treatment Mean age (years): I1=54.2 I2=53.7 C=53.8 Gender: I1=37% female I2=33% female C=33% female	I1=**CBT**: Cognitive-behavioural treatment (8 week) led by a social worker with more than 10 years of group counselling experience. Group sessions lasted 1 h per week and had six to nine participants. The focus was on cognitive and behavioural strategies to reduce maladaptive anxiety and depression (*n*=29) I2=**Social support**: Social support (8 week) led by a social worker with more than 10 years of group counselling experience. Group sessions lasted 1 h per week and had six to nine participants. The leader encouraged members to describe their feelings about having cancer, to identify shared problems, to discuss how these are handled and to adopt supportive roles (*n*=23) C=No treatment control (*n*=26)	Depressive symptoms CES-D and SCL-90-R measured at baseline, 8 weeks and 6 months	Subjects who received either cognitive behavioural or social support group interventions had significantly lower CES-D scores than controls at 8 weeks (*P*<0.01) and at 6 months social support group interventions had significantly lower CES-D scores than controls (*P*<0.01). Mean decrease in CES-D score for patients from baseline to 6 months was 11% for the cognitive behavioural group, 29% for the social support group and 14% in the control group	Not reported

Winzelberg *et al* (2003), USA Design: Experimental randomised controlled trial Random allocation to treatment groups: YES Concealed treatment allocation: NO Blinding of investigators: NO Blinding of subjects: NO Known confounders accounted for by study design: NO, cointerventions not reported Attrition of subjects and reasons: YES Dropout rate: 19% overall	72 women with primary breast carcinoma Mean age (years) (s.d.): 49.5 (6.2) There were no baseline differences between groups	I=**Social support/education**: web-based psychoeducational support group, 12 weeks. The program introduced a new topic related to breast cancer each week and the mental health professional facilitated a discussion on these topics and related concerns. On the website participants were able to read personal stories from survivors, share their own experiences and keep a private web-based personal journal. Participants wrote a brief description of how they were feeling when they logged on (*n*=36) C=Wait-list control (*n*=36)	Depressive symptoms CES-D Measured at baseline and at end of intervention (12 weeks)	CES-D (*P*<0.01), ES=0.54 Mean decrease in CES-D score for patients from baseline to 12 weeks was 36% in the experimental group and 4% in the control group	Not reported

*Psychotherapeutic studies (breast cancer)*					
Antoni *et al* (2001), USA Design: Experimental randomised controlled trial Random allocation to treatment groups: YES Concealed treatment allocation: NO Blinding of investigators: NO Blinding of subjects: NO Known confounders accounted for by study design: NO, cointerventions not reported Attrition of subjects and reasons: attrition but not reason: Dropout rate: 26% overall	100 women newly treated for stage 0–II breast cancer Mean age (years) (s.d.): I=48.1 (9.0) C=52.1 (9.0)	I=**CBT**: 10-week group cognitive behavioural stress management intervention. Weekly for ten 2-h sessions. It included both problem-focused (e.g. active coping and planning) and emotion-focused (e.g. relaxation training, use of emotional support) coping strategies (*n*=47) C=**Education/information**: One-day group seminar approximately 16–18 weeks postsurgery. To provide at least some information on all of the topics covered by the intervention condition (*n*=53)	Depressive symptoms POMS, CES-D Measured at baseline, post-intervention (3 months), 3 months and 9 months	POMS, NS CES-D In the intervention group the proportion of women meeting criteria for moderate levels of depressive symptoms fell significantly at 3 months postintervention, 6 months and 12 months (*P*<0.04)	Not reported

Goodwin *et al* (2001), Canada Design: Experimental randomised controlled trial Random allocation to treatment groups: YES Concealed treatment allocation: YES Blinding of investigators: YES Blinding of subjects: NO Known confounders accounted for by study design: PARTIAL Attrition of subjects and reasons: YES Dropout rate: I=30%, C=35%, NS	235 women with metastatic breast cancer who were expected to survive at least 3 months Mean age (years) (s.d.): I=49.5 (8.4) C=51.5 (10.3)	I=**CBT**: Weekly *supportive–expressive* group therapy (90 min sessions) for at least one year. Groups consisted of 8–12 women and two leaders. A monthly 90 min session was provided for family and friends (*n*=158) C=**Education/information**: Education materials only. No supportive–expressive group therapy intervention (*n*=77)	Depressive symptoms POMS Measured at baseline and 4, 8 and 12 months after randomisation	POMS Depression dejection was significantly lower in the intervention group (*P*=0.002) 1 year after randomisation The psychological intervention was not associated with prolonged survival	Not reported

*Psychotherapeutic studies (breast cancer)*					
Classen *et al* (2001), USA Design: Experimental randomised controlled trial Random allocation to treatment groups: YES Concealed treatment allocation: NO Blinding of investigators: NO Blinding of subjects: NO Known confounders accounted for by study design: NO, cointerventions not reported Attrition of subjects and reasons: YES Dropout rate: 18% overall	125 women with metastatic breast cancer Mean age (years) (s.d.): I=52.9 (10.7) C=54.0 (10.7)	I=**CBT/education**: 1 year of weekly (90 min sessions) of supportive–expressive group therapy and educational materials. The treatment strategy is to facilitate discussion of issues that are uppermost in the patients’ minds rather than imposing topics to be discussed (*n*=64) C=**Education/information**: Education materials only (*n*=61)	Depressive symptoms POMS Measured at baseline and every 4 months during the first year	POMS Depression When follow-up assessments undertaken within 1 year of the patients’ death were excluded in the secondary analyses POMS Depression subscale was significantly lower for the treatment group, ES=0.27	Not reported

Fukui *et al* (2000), Japan Design: Experimental randomised controlled trial Random allocation to treatment groups: YES Concealed treatment allocation: NO Blinding of investigators: NO Blinding of subjects: NO Known confounders accounted for by study design: NO, cointerventions not reported Attrition of subjects and reasons: YES Dropout rate: 8% overall	50 women with primary breast carcinoma Mean age (years) (s.d.): I=52.6 (6.8) C=54.3 (7.5)	I=**CBT**: Six group sessions of structured psychosocial intervention with a cognitive-behavioural approach. It included health education, coping skills training, stress management and psychological support (*n*=25) C=Wait-list control (*n*=25)	Depressive symptoms HADS, POMSMeasured at baseline, 6 weeks and 6 months	HADS, NS	Not reported

Sandgren *et al* (2000), USA Design: Experimental randomised controlled trial Random allocation to treatment groups: YES Concealed treatment allocation: NO Blinding of investigators: NO Blinding of subjects: NO Known confounders accounted for by study design: NO, cointerventions not reported Attrition of subjects and reasons: YES Dropout rate: 15% overall	53 women newly diagnosed with stage I or II breast cancer Mean age (years) (s.d.): 51.23 (12.5)	I=**CBT**: Individual psychosocial therapy (cognitive behavioural) delivered by three female psychology graduate students by telephone. There were a total of ten therapy sessions (average duration 20–25 min) once a week for 4 weeks and then every other week for six more sessions. The intervention focused on four areas: providing support, teaching coping skills, managing anxiety and stress and helping to solve patient generated problems.(*n*=24) C=No therapy control (*n*=29)	Depressive symptoms POMS Measured at baseline, 1, 4 and 10 months	POMS Depression, NS	Not reported

*Psychotherapeutic studies (breast cancer)*					
Edelman *et al* (1999), Australia Design: Experimental randomised controlled trial Random allocation to treatment groups: YES Concealed treatment allocation: NO Blinding of investigators: NO Blinding of subjects: NO Known confounders accounted for by study design: NO, cointerventions not reported Attrition of subjects and reasons: Attrition but no reasons Dropout rate: 23% overall	124 women with metastatic breast cancer Mean age (years) (range): 50 (29–65) There were no significant differences in age between groups	I=**CBT**: Group cognitive behaviour therapy. Eight weekly sessions of group CBT followed by a family night and three further monthly sessions. The therapy incorporated the use of cognitive and behavioural techniques as well as encouraging the expression of feeling and building of group support (*n*=62) C=No therapy control (*n*=62)	Depressive symptoms POMS Measured at baseline, after completion of therapy, 3 months and 6 months	After completion of therapy significant improvement in depressive symptoms (*P*=0.008)	Not reported

Marchioro *et al* (1996), Italy Design: Experimental randomised controlled trial Random allocation to treatment groups: YES Concealed treatment allocation: NO Blinding of investigators: NO Blinding of subjects: NO Known confounders accounted for by study design: NO, cointerventions not reported Attrition of subjects and reasons: NO	36 newly diagnosed women with non-metastatic breast cancer assigned to adjuvant chemotherapy after surgery Median age (years): I=53 C=52	I=**Counselling/psychotherapy**: Weekly 50 min individual cognitive individual psychotherapy and bimonthly family counselling with a psychologist. Cognitive psychotherapy was aimed at analysis and relief of major problems related to cancer diagnosis and therapy: anxiety and/or depression, loss of behavioural and emotional control, altered cognitive functioning, social and role limitations and physiological symptoms (*n*=18) C=Standard follow-up (*n*=18)	Depressive symptoms BDI Measured at baseline and after 1, 3, 6 and 9 months	Depression scores improved significantly over time in the intervention group (ES=0.27)	Not reported

McArdle *et al* (1996), UK Design: Experimental randomised controlled trial Random allocation to treatment groups: YES Concealed treatment allocation: NO Blinding of investigators: NO Blinding of subjects: NO Known confounders accounted for by study design: NO, cointerventions not reported Attrition of subjects and reasons: YES Dropout rate: 17% overall	272 women undergoing surgery for breast cancer Median age (years): I1=59 I2=55 I3=56 C=57	I1=routine care plus **education/information/counselling/support from breast care nurse**: routine care (including information booklet) plus support from breast care nurse (information and counselling) (*n*=70) I2=routine care plus **education/information/counselling/support from voluntary organisation** routine care plus support from voluntary organisation (offering three types of support: information, counselling and regular group meetings with fellow cancer suffers) (*n*=66) I3=**routine care plus education/information/counselling/support** **from nurse and organisation** (*n*=69) C=routine care from ward staff (*n*=67)	Depressive symptoms GHQ-28, HADS Measured at 1, 3, 6 and 12 months after surgery	Scores were lower in patients offered support from breast care nurse alone (I1) GHQ-28 (*P*=0.015) HAD Depression (*P*=0.003) Severe depression (*P*=0.072)	Not reported

*Psychotherapeutic studies (gynaecological cancer)*					
Burton *et al* (1995), UK Design: Experimental randomised controlled trial Random allocation to treatment groups: YES Concealed treatment allocation: NO Blinding of investigators: NO Blinding of subjects: NO Known confounders accounted for by study design: NO, cointerventions not reported Attrition of subjects and reasons: Attrition unclear and no reasons	200 women awaiting mastectomy or sector mastectomy for breast cancer Mean age (years): I1=61 I2=62 I3=64 C=57	I1=**Counselling/psychotherapy**: Individual preoperative interview and a 30 min brief psychotherapeutic intervention (*n*=50) I2=Individual preoperative interview and a 30 min ‘chat’ to control for the effects of attention (*n*=50) I3=preoperative interview only (*n*=50) C=routine hospital care control (*n*=50)	Depression, Depressive symptoms HADS, GHQ-28, PSE schedule Measured at baseline, 4 days, 3 months and 1 year after surgery		More control patients were cases for depression on PSE criteria at one year than patients in the experimental groups (*P*=0.037)

Petersen and Quinlivan (2002), Australia Design: Experimental randomied controlled trial Random allocation to treatment groups: YES Concealed treatment allocation: YES Blinding of investigators: NO Blinding of subjects: NO Known confounders accounted for by study design: NO, cointerventions not reported Attrition of subjects and reasons: YES Dropout rate: 6% overall	53 patients with gynaecological cancer Mean age (years) (s.d.): I=63.0 (9.6) C=61.2 (13.5)	I=**Counselling/relaxation**: 1 h relaxation and counselling interview performed by a senior medical practitioner involved in the patient's care. The structured interview consisted of listening to a tape of relaxation music for approx. 5 min followed by a series of relaxation exercises that lasted 20 min. Patients were encouraged to use the techniques at home if they felt stressed or anxious. The final 30–35 min was spent discussing the patient's condition (*n*=27) C=Patients were provided with all the normal postoperative support, counselling and information services provided by the respective hospitals (*n*=26)	Depressive symptoms**/**depression HADS GHQ-28 Measured at baseline and six weeks	The intervention was associated with a significant reduction in total HADS score (*P*=0.002), reduction in HADS Mild/moderate Depression subscale (*P*=0.02), and lower GHQ-28 scores (*P*=0.03) No significant difference was found in the fourth subscale of major depression	HADS score case/noncase (*P*=0.0001)

*Psychotherapeutic studies (testicular cancer)*					
Moynihan *et al* (1998), UK Design: Experimental randomised controlled trial Random allocation to treatment groups: YES Concealed treatment allocation: YES Blinding of investigators: YES Blinding of subjects: NO Known confounders accounted for by study design: NO, cointerventions not reported Attrition of subjects and reasons: YES Dropout rate: 7% overall	73 men with newly diagnosed testicular cancer	I=**CBT/PST**: Adjuvant psychological therapy which uses both cognitive and behavioural approaches includes strategies such as problem solving and regaining control. The intervention consisted of six individual sessions each lasting 1 h by a state registered mental health nurse who was experienced in caring for patients with testicular cancer (*n*=36) C=standard care (*n*=37)	Depression/depressive symptoms HADS Measured at baseline 2, 4 and 12 months	HAD Depression scale, NS	The proportion of patients scoring above the threshold on subscale was not influenced by adjuvant psychological therapy

*Prostate cancer*					
Weber *et al* (2004), USA Design: Experimental randomised controlled trial Random allocation to treatment groups: YES Concealed treatment allocation: NO Blinding of investigators: NO Blinding of subjects: NO Known confounders accounted for by study design: PARTIAL Attrition of subjects and reasons: YES Dropout rate: 0%	30 men who had recently undergone a radical prostatectomy for prostate cancer Mean age (years) (s.d.): I=57.5 (6.7) C=59.7 (6.6)	I=**Social support:** Peer support program – 10 men who were long term survivors of prostate cancer received 2 h training session to act as support partners. Each dyad met eight times during an 8 week period and each dyad determined the focus and direction of its own exchanges (*n*=15) C=usual care (*n*=15)	Depressive symptoms GDS short versionAssessed at baseline, 4 weeks and 8 weeks	There was a significant difference in depression between groups at 4 weeks (*P*=0.02) (ES=0.99), but no significant difference at 8 weeks Mean decrease in GDS score for patients from baseline to 4 weeks was 88% in the experimental group and 0% in the control group	Not reported


BZSDS=Brief Zung Self-Rating Depression Scale; HAM-D21=21-item Hamilton Depression Rating Scale; SSRI=selective serotonin reuptake inhibitor; CES-D=Center for Epidemiological Studies Depression scale; POMS=Profile of Mood States; DD=Depression subscale of the POMS; TCA=tricyclic antidepressant; MADRS=Montgomery Asberg depression rating scale; CDS=Carroll Depression Scale; DSM-IV=Diagnostic and Statistical Manual of Mental Disorders IV; DSM III=Diagnostic and Statistical Manual of Mental Disorders III; HADS=Hospital Anxiety and Depression Scale; CESD-20=20-item Center of Epidemiological Studies Depression Scale; ES=effect size; Dutch POMS=Dutch version of the shortened Profile of Mood States; GDS short versio*n*=Geriatric Depression Scale; BDI=Beck Depression Inventory; GHQ-28=28-item General Health Questionnaire; GHQ-30=30-item General Health Questionnaire; SCL-90-R=Symptom Checklist 90 Revised; s.d.=standard deviation; PST=problem-solving therapy; CBT=cognitive behaviour therapy; PSE schedule=Present State Examination schedule; CNQ=Cancer Needs Questionnaire – short form; EORTC QLQ-30=European Organisation for Research and Treatment of Cancer Quality of Life Questionnaire C30.

**Table 2:** Methodological quality of included trials summary (*n*=24)

	**No. of pharmacological trials (%)**			**No. of psychotherapeutic trials (%)**		
**Quality indicator**	**Yes, fulfilled**	**No**	**Unclear**	**Yes, fulfilled**	**No**	**Unclear**
Random allocation to treatment groups – method sufficiently described	6 (100%)	—	—	18 (100%)	—	—
Blinding of investigators	5 (83%)	1 (17%)	—	2 (11%)	16 (89%)	—
Blinding of subjects	6 (100%)	—	—	—	18 (100%)	—
Concealed treatment allocation	3 (50%)	—	3 (50%)	6 (33%)		12 (67%)
						
*Groups similar at baseline*						
Gender	5 (83%)	1 (17%)	—	17 (94%)	—	1 (6%)
Age	6 (100%)	—	—	13 (72%)	2 (11%)	3 (17%)
Ethnicity	3 (50%)	—	3 (50%)	6 (33%)	—	12 (67%)
Disease distribution	3 (50%)	—	3 (50%)	15 (83%)	1 (6%)	2 (11%)
Cancer treatment	3 (50%)	1 (17%)	2 (33%)	10 (55%)	1 (6%)	7 (39%)
Depression/depressive symptoms	6 (100%)	—	—	15 (83%)	—	3 (17%)
Screened for depression	4 (67%)	2 (33%)	—	2 (11%)	16 (89%)	—
						
*Cointerventions*						
Psychotherapeutic cointerventions avoided by trial design	—	6 (100%)	—	2 (11%)	12 (67%)	4 (22%)
Psychotherapeutic cointerventions monitored	—	6 (100%)	—	5 (28%)	9 (50%)	4 (22%)
Pharmacological cointerventions avoided by trial design	5 (83%)	1 (17%)	—	—	18 (100%)	—
Pharmacological cointerventions monitored	1 (17%)	5 (83%)		—	18 (100%)	—
CAM interventions for depression avoided by trial design	—	6 (100%)	—	—	18 (100%)	—
CAM interventions for depression monitored	—	6 (100%)	—	—	18 (100%)	—
						
Clear description of intervention	6 (100%)	—	—	18 (100%)	—	—
Outcome measures valid/reliable	6 (100%)	—	—	18 (100%)	—	—
Reporting caseness for depression at follow-up	3 (50%)	3 (50%)	—	5 (28%)	13 (72%)	—
						
*Withdrawal*						
Attrition of subjects reported	6 (100%)	—	—	16 (89%)	2 (11%)	—
Reasons reported	6 (100%)	—	—	13 (72%)	5 (28%)	—
Sample size justification before study	4 (67%)	2 (33%)	—	4 (22%)	14 (78%)	—
*N*</=100	2 (33%)	4 (67%)	—	9 (50%)	9 (50%)	—
Intention-to-treat analyses stated	3 (50%)	3 (50%)	—	6 (33%)	12 (67%)	—

**Box 1:** Search strategy terms

Search terms were taken from known articles relevant to the review and the Medical Subject Headings (MeSH) thesaurus of the US National Library of Medicine (NLM). The search terms included: ‘*Depression*’; ‘*Depressive Disorder*’; ‘*Depressive Disorders*’; ‘*Disorder, Depressive*’; ‘*Disorders, Depressive*’; ‘*Neurosis, Depressive*’; ‘*Depressive Neuroses*’; ‘*Depressive Neurosis*’; ‘*Neuroses, Depressive*’; ‘*Melancholia*’; *‘Melancholias*’; ‘*Unipolar Depression*’; ‘*Depression, Unipolar*’*; ‘Depressions, Unipolar*’; ‘*Depression, Endogenous*’; ‘*Depressions, Endogenous*’; ‘*Endogenous Depression’;* ‘*Endogenous Depressions*’; *‘Depressive Syndrome*’; ‘*Depressive Syndromes*’; ‘*Syndrome, Depressive*’; ‘*Syndromes, Depressive*’; ‘*Depression, Neurotic*’; ‘*Depressions, Neurotic’*; ‘*Neurotic Depression*’; ‘*Neurotic Depressions*’; ‘*Depressions’*; ‘*Depressive Symptoms*’; ‘*Depressive Symptom*’; ‘*Symptom, Depressive*’; *‘Symptoms, Depressive*’; ‘*Emotional Depression*’; ‘*Depression, Emotional*’; *‘Depressions, Emotional*’; ‘*Psychiatric Morbidity*’; ‘*Psychological Morbidity*’; *‘Cancer*’; ‘*Neoplasms*’; *‘Neoplasms, Second Primary*’; ‘*Hypothalamic Neoplasms*’; *‘Brain Neoplasms*’; ‘*Brain Stem Neoplasms*’; *‘Head and Neck Neoplasms*’; ‘*Spinal Cord Neoplasms*’; *‘Meningeal Neoplasms*’; ‘*Urologic Neoplasms*’*; ‘Cerebellar Neoplasms*’; ‘*Supratentorial Neoplasms*’; *‘Unrogenital Neoplasms*’; ‘*Sigmoid Neoplasms*’; ‘*Infratentorial Neoplasms*’; ‘*Cerebral Ventricle Neoplasms*’*; ‘Testicular Neoplasms*’; ‘*Pharyngeal Neoplasms*’; ‘*Pelvic Neoplasms*’; *‘Ovarian Neoplasms*’; ‘*Breast Neoplasms*’; ‘*Antidepressive Agents’*; ‘*Antidepressive Agents – adverse effects*’; ‘*Serotonin Uptake Inhibitors*’*; ‘Fluoxetine*’; ‘*Cognitive Therapy*’; *‘Randomised Controlled Trials*’

**Table A1:** 

Reviewer _________				
Article _________				
1. Study design (choose 1 only):				
Experimental randomised:				
______ Placebo-controlled trial				
______ Controlled trial				
______ Comparative trial, no placebo				
______ Time series trial				
______ Crossover trial				
Experimental, unrandomised:				
______ Placebo controlled trial				
______ Comparative trial, no placebo				
______ Time series trial				
______ Crossover trial				
______ Natural experiment				
Nonexperimental:				
______ Cohort, prospective				
______ Cohort, retrospective				
______ Cross-sectional				
______ Case–control				
______ Case reports or case series				
______ None of the above (describe):				
				
2. What was the study question? (please use space below)				
	Yes	Partial	No	N/A
3. Was the study question sufficiently described?				
4. Was the study design appropriate to answer the study question?				
5. Were both inclusion and exclusion criteria specified? (If case study, check N/A)				
6. For case studies only: Were patient characteristics adequately reported? (If not case study, check N/A.)				
7. Were subjects appropriate to the study question?				
8. Were control subjects appropriate? (If no controls were used, check No).				
9. Were subjects randomly selected from the target population?				
10. If subjects were randomly selected, was the method of random selection sufficiently well described? (If subjects were not randomly selected, check N/A).				
11. If subjects were randomly allocated to treatment groups, was the method of random allocation sufficiently described? (If subjects were not randomly allocated, check N/A).				
12. If blinding of investigators to intervention was possible, was it reported? (If not possible check N/A).				
13. If blinding of subjects to intervention was possible, was it reported? (If not possible, check N/A).				
14. Was measurement bias accounted for by methods other than blinding?				
15. Were known confounders accounted for by study design? (If no known confounders, check N/A).				
16. Were known confounders accounted for by analysis? (If no known confounders, check N/A).				
17. Was there a sample size justification before the study?				
18. Were post hoc power calculations or confidence intervals reported for statistically nonsignificant results?				
19. Were statistical analyses appropriate?				
20. Were statistical tests stated?				
21. Were exact P values or confidence intervals reported for each test?				
22. Were attrition of subjects and reason for attrition recorded?				
23. For those subjects who completed the study, were results completely reported?				
24. Do the findings support the conclusions?				

**Table A2:** 

**Reviewer:**	**Authors/date**	**Country of origin:**		
**Study details**		Yes	No	Unclear
**Patient groups similar at baseline? No sig. diffs.**	Gender			
Age				
Ethnicity				
Disease distribution				
Cancer treatment				
Depression/Depressive symptoms				
**Setting/s**				
**Screening/diagnostic procedure for depression or depressive symptoms**				
(consider issues for access to healthcare treatment)				
Intervention treatment				
1. Description of intervention and implementation?				
2. Measure of compliance?				
3. Cointerventions: avoided by trial design/monitored				
(Also consider issues for access to healthcare treatment)				
**Possible adverse effects**				
**Use of diagnostic criteria for depression?**				
Outcome measures used (adequately described, valid/reliable?)				
**Dropout rates and reasons for withdrawal**				
**Noneligible patients**				
See table				
**Data analysis**		Yes	No	Unclear
Economic analysis as part of trial?				
**Conclusion**				

## Appendix A

Data Extraction Form Methodological Quality Instrument (Cho and Bero, 1994) (Table A1).

Data Extraction Form: Pharmacological intervention/psychosocial intervention (Table A2).
